# Premature mortality in young people accessing early intervention youth mental healthcare: data-linkage cohort study

**DOI:** 10.1192/bjo.2023.43

**Published:** 2023-04-24

**Authors:** Catherine M. McHugh, Frank Iorfino, Natalia Zmicerevska, Yun Ju Christine Song, Adam Skinner, Elizabeth M. Scott, Ian B. Hickie

**Affiliations:** Brain and Mind Centre, University of Sydney, Sydney, NSW, Australia; and Discipline of Psychiatry, University of New South Wales, Sydney, NSW, Australia; Brain and Mind Centre, University of Sydney, Sydney, NSW, Australia; Brain and Mind Centre, University of Sydney, Sydney, NSW, Australia; and School of Medicine, University of Notre Dame Australia, Sydney, NSW, Australia

**Keywords:** Youth mental health, suicidal behaviour, deliberate self-harm, mortality, accident and injury

## Abstract

**Background:**

Understanding premature mortality risk from suicide and other causes in youth mental health cohorts is essential for delivering effective clinical interventions and secondary prevention strategies.

**Aims:**

To establish premature mortality risk in young people accessing early intervention mental health services and identify predictors of mortality.

**Method:**

State-wide data registers of emergency departments, hospital admissions and mortality were linked to the Brain and Mind Research Register, a longitudinal cohort of 7081 young people accessing early intervention care, between 2008 and 2020. Outcomes were mortality rates and age-standardised mortality ratios (SMR). Cox regression was used to identify predictors of all-cause mortality and deaths due to suicide or accident.

**Results:**

There were 60 deaths (male 63.3%) during the study period, 25 (42%) due to suicide, 19 (32%) from accident or injury and eight (13.3%) where cause was under investigation. All-cause SMR was 2.0 (95% CI 1.6–2.6) but higher for males (5.3, 95% CI 3.8–7.0). The mortality rate from suicide and accidental deaths was 101.56 per 100 000 person-years. Poisoning, whether intentional or accidental, was the single greatest primary cause of death (26.7%). Prior emergency department presentation for poisoning (hazard ratio (HR) 4.40, 95% CI 2.13–9.09) and psychiatric admission (HR 4.01, 95% CI 1.81–8.88) were the strongest predictors of mortality.

**Conclusion:**

Premature mortality in young people accessing early intervention mental health services is greatly increased relative to population. Prior health service use and method of self-harm are useful predictors of future mortality. Enhanced care pathways following emergency department presentations should not be limited to those reporting suicidal ideation or intent.

Longitudinal studies of cohorts accessing mental health services have found increased risk of premature mortality due to suicide, as well as deaths attributable to accident and injury, substance use and cardiometabolic disease.^1,2^ In health systems globally, an increased focus on early intervention has led to new cohorts of young people accessing mental health services. Youth mental health cohorts experience high rates of suicidal ideation, thoughts and behaviour (SITB) and non-suicidal self-harm, as well as increased rates of other risk factors associated with increased mortality across the lifetime, such as alcohol and substance use, poorer physical health and higher rates of accident and injury. Although previous register-based studies of hospital and out-patient populations have found that young people with psychiatric disorders are at increased risk of premature mortality, it is not clear whether this elevated risk is present in youth cohorts accessing community-based early intervention mental health services, who are typically considered to be at earlier stages of illness than those accessing hospital-based or out-patient mental health services.^3^

Suicide remains a leading cause of death of young people in the population,^4^ and many countries have reported increased demand for mental health services from young people experiencing SITB and non-suicidal self-harm. Increased demand has been evident in youth mental health services and in emergency departments, where rates of suicidal ideation and/or behaviour and non-suicidal self-harm-related presentations have been increasing.^5–7^ Given that previous suicidal behaviour remains one of the strongest risk factors for future suicidal behaviour,^8,9^ these presentations are a compelling target of secondary intervention for health services.

Yet how clinical presentations for suicidal ideation and/or behaviour or non-suicidal self-harm relate to mortality risk from suicide and other causes varies both between and within populations. In the UK, cohort studies of adults presenting to emergency departments with self-harm, which includes both suicide attempts and self-harm without suicidal intent, have shown greatly increased risks of premature mortality from related causes of death, such as suicide or accident and injury, as well as other physical health causes relative to the general population.^2^ Over a median of 6 years follow-up, all-cause mortality risk was elevated in males (standardised mortality ratio (SMR) 4.1, 95% CI 3.8–4.3) and females (3.2, 2.9–3.4), with accidental deaths being associated with the greatest increase in mortality risk.^2^ Hawton et al (2020) found a similarly increased risk of premature mortality in a cohort of children and adolescents presenting to emergency departments with self-harm, also defined to include both suicide attempts and non-suicidal self-harm.^10^ In both cohorts, male sex was associated with almost twice the absolute risk of premature mortality. However, female sex was still associated with a greater increase in mortality risk relative to females in the general population. In both cohorts, older age at the time of presentation for non-fatal self-harm predicted greater risk of premature mortality.

A detailed understanding of cause-specific mortality risk and predictors of premature mortality within a population is essential for delivering effective clinical and population-level interventions. Characterising health service use prior to death may also identify opportunities for targeted interventions relevant to youth mental health cohorts. For example, a history of psychiatric admission has been shown to increase risk of premature mortality from suicide and other causes, with the greatest risk of suicide occurring soon after discharge.^11^ On the other hand, the risk of premature death from other causes, including physical health causes, increases over time. Other factors known to moderate the risk of mortality following presentations for SITB or self-harm include the method of self-harm used, a diagnosis of major mental illness or alcohol or substance use disorder, and a history of repeat presentations with self-harm.^12^

The first aim of this study was to estimate premature mortality risk in a cohort of young people accessing early intervention youth mental healthcare by determining SMRs as well as all-cause and cause-specific mortality rates. Mortality from suicide, accident or injury and other physical health causes was included. The second aim was to establish the most common primary and contributing causes of death. Finally, the study aimed to identify whether prior health service use is a useful predictor of death from suicide or accidental and injury.

## Method

### Participants and setting

The study used data from the Brain and Mind Research Register (BPRR), a longitudinal cohort study of 7081 young people presenting to the youth mental health clinics of the Brain and Mind Centre in the Sydney suburbs between October 2008 and October 2020.^13,14^ The early intervention clinics consist of primary mental healthcare (headspace) and more specialised mental health services, including psychiatrists and psychologists. They are accessed by young people aged 12–30 years old, presenting at early clinical stages (non-specific symptoms) through to full-threshold disorders. The study included young people aged 12–30 years to reflect the cohort of young people accessing care via headspace services.

Inclusion criteria for the BPRR were: (a) age between 12 and 30 years at first presentation to the service, and (b) attendance of at least one visit to the service. Exclusion criteria for the BPRR were: (a) medical instability or lack of capacity to give informed consent (as determined by a psychiatrist), (b) history of neurological disease (e.g. tumour, head trauma, epilepsy), (c) medical illness known to affect cognitive and brain function (e.g. cancer, electroconvulsive therapy in the past 3 months), (d) clinically evident intellectual disability, and/or (e) insufficient English to participate in the research protocol.

### Data linkage

The BPRR was linked to three external databases held by the Centre for Health Record Linkage in New South Wales (NSW), Australia.^14,15^ These databases were the Register of Births, Deaths and Marriages (RBDM), the Australian Coordinating Registry Cause of Death Unit Record File (ACR-COD URF), the Emergency Department Data Collection (EDDC) and the Admitted Patient Data Collection (APDC). Gender and age were selected from the BPRR. Other exposures of interest, such as emergency department presentations and hospital admissions, were selected from the EDDC and APDC. The EDDC uses diagnostic codes primarily from Systematized Nomenclature of Medicine Clinical Terms (SNOMED CT) but also from the ICD-9-CM, ICD-10-CM and ICD-10-AM. In Australia, emergency department diagnostic codes are applied by clinicians at the point of care rather than by professional clinical coders. Clinicians begin by entering diagnostic terms (or the presenting problem) as free text, with the closest matching SNOMED CT term then presented for selection. As a result, diagnostic codes tend to reflect presenting symptoms rather than true diagnoses.^16^ This has limited consequences for the aims of the current study, as psychiatric diagnoses are made formally only by specialised services, often over a number of visits. The current study aimed to capture broad categories of emergency department presentations related to outcomes of interest. Two authors (C.M.M. and N.K.) with clinical training in emergency department care and mental health independently reviewed the diagnostic terms and grouped presenting symptoms into several predetermined categories including (a) suicidal ideation, behaviour or self-harm; (b) accident and injury; (c) drug and alcohol use; (d) medical or physical illness; or (e) other. Interrater reliability was 98%. Any discrepancies between coders were resolved via consensus. Only one reason for presentation was recorded for each emergency department presentation.

This study defined suicidal behaviour and non-suicidal self-harm in clinical populations of young people as occurring along a spectrum of intent and lethality, in keeping with previous definitions by Hawton et al.^10^ Self-harm is used to describe an intentional act associated with an intent to harm oneself, whether or not it was associated with an intent to end one's life. This approach to combining suicidal behaviour and non-suicidal self-harm was used given that the expression (and recording) of intent associated with an act of self-harm in emergency department settings may be influenced by factors including an individual's desire to avoid restrictive interventions, therapeutic rapport and/or clinician skill. The EDDC also does not require clinicians to formally differentiate between these constructs. Given these factors, the use of the broader construct of suicidal behaviour and self-harm was considered more valid than the use of specific terms such as non-suicidal self-injury, which at present are not typically used in clinical settings in Australia and thus are not likely to be accurately recorded. Intentional self-poisoning was included as a subtype of self-harm. Suicidal behaviour refers to self-harm associated with any suicidal intent. In categorising the primary diagnostic terms associated with an emergency department presentation, the suicidal ideation, behaviour or self-harm category was applied if the terms intentional self-injury or intentional self-poisoning were used. Any self-poisoning which was specifically labelled as accidental was included in the ‘accident and injury’ category. Many self-poisoning presentations were not specifically labelled as intentional or accidental. In such instances, poisoning or overdose with known drugs of misuse or recreational drugs were categorised as ‘drug and alcohol’ presentations. Pharmaceutical drug poisoning or overdose, where patterns of misuse were not accepted to have a recreational component, were considered ‘suicidal or self-harm’ presentations.

Variables of interest selected from the APDC were mental health unit (psychiatric) admissions and intensive care unit (ICU) admissions. ICU refers to admissions to the general hospital ICU, not mental health admissions. ICU admissions were included as an exposure of interest given their association with high-risk suicidal behaviour and increased mortality. The total numbers of admissions and emergency department presentations per individual were counted, as well as lifetime history of each category of emergency department presentation. Childhood presentations (age <12 years) to an emergency department for any mental health reason (e.g. SITB, other psychiatric symptoms, or drug- and alcohol-related) were included in determining lifetime history of these presentation types; however, for presentations related to medical or physical illness or common injuries, lifetime history was defined as over the age of 12 years. Emergency department presentations and hospital admissions that occurred on the same date as death were excluded.

### Outcome measures

The primary outcome measure was mortality from all causes and mortality due to suicide or accident. Date of death was extracted from the NSW RBDM. Underlying cause of death and contributing causes were extracted from the ACR-COD URF. The ACR-COD URF uses ICD-10 codes only. Deaths were considered suicides if coded as intentional (X60–X84) or self-inflicted but with undetermined intent (Y10–Y34), in keeping with the convention used within the suicide research literature.^10,17^ Deaths where cause had not yet been allocated an ICD code within the ACR-COD URF were included as suicide or accident deaths, as according to the ACR-COD URF a delay in cause of death being assigned indicates a coronial investigation is required to determine a specific cause of an unnatural death (i.e. suicide, accident or injury-related).^18^ Therefore, these deaths without an ICD code were analysed with suicide and accident or injury deaths.

Suicide deaths were further subcategorised by method, including self-injury (ICD-10 codes X70–X84, Y20–Y34) and intentional self-poisoning (X61–X69, Y11–Y19). Accidental deaths (V01–X59) were subcategorised into accidental poisoning (X40–X49), transport accidents (V01–X59) or other external causes of accidental injury (W00–X59). Contributing causes of death are presented where they are distinct from the primary cause of death and are referred to as secondary causes.

Ethics approval for the BPRR was granted by the University of Sydney Human Research Ethics Committee (2012/1626). Participants in the BPRR consented to their routinely collected clinical information being used for clinical research for a period of 5 years and to being contacted about additional research. The BPRR ethics approval (2012/1626) stipulated that additional consent would be necessary if the participant was required to return to the clinic for additional assessment for research. The current study did not require any additional assessments or further contact with participants. Ethics approval for the current study linking the BPRR to external data-sets was granted by the NSW Population and Health Services Research Ethics Committee (PHSREC) (2019/ETH12201). A waiver of consent was granted by the PHSREC as the following National Statement on Ethical Conduct in Human Research (2007) criteria were met: (a) the purposes of the study could not be met without temporary reidentification; (b) contacting participants for reconsent was not practicable, given the large number of participants and that the key outcome of interest was death; and (c) there was no reason to believe participants would not have consented to the use of their routinely collected data for this study, given they had previously consented to more sensitive information being accessed, such as their individual medical records.

### Analysis

#### Mortality rates and SMR

SMR was calculated using indirect methods of standardisation by age category (15–19, 20–24, 25–29, 30–34, 35–39, 40–45 years) and sex. The group used to determine the expected number of deaths (the denominator) was the general population of NSW in the year that marked the mid-point of the study.^19^ Mortality rates were calculated using the person-time method, which is the number of deaths divided by the person-years of follow-up. For calculation of the SMR, the date of the individual's first health service visit after the age of 12 years old was used to calculate years of follow-up. If the date of the individual's first youth mental health clinic visit was missing, these cases were omitted from the calculation of years of follow-up. Mortality rates for specific causes of death, including suicide, accident and injury, and those relating to medical illness and where cause was under investigation were also calculated using this method.

#### Cox regression

For survival analysis, the follow-up time was calculated from the date of the last health visit (youth mental health clinic visit, emergency department presentation, mental health unit admission or ICU admission) to the date of death or the end of the study. Where the first youth mental health clinic date was missing and there was no history of other health visit types, it was not possible to accurately calculate follow-up time; thus, these individuals were not included in the Cox regression.

Cox proportional hazards regression modelling was conducted for emergency department presentations and hospital admissions, adjusted for gender. Exposure variables of interest were gender and lifetime history of emergency department presentation for suicidal ideation or behaviour, or mental health, drug and alcohol, accident and injury or medical reasons. Hospital admission variables of interest included lifetime history of mental health unit admission or ICU admission. Modelling was conducted first with all-cause mortality as the outcome and subsequently with deaths due to suicide, accident and injury, or cause under investigation as the outcome of interest. Partial residuals were plotted in order to check that proportional hazards were constant over time.

### Sensitivity analysis

A sensitivity analysis was conducted to assess the effects of different constructs of self-poisoning on the prediction of mortality outcomes. In the sensitivity analysis, self-poisoning presentations were considered as distinct from other forms of self-harm. All types of self-poisoning, whether intentional, accidental or of unknown intent, were considered as one category distinct from other types of accident, injury or drug and alcohol presentations. Thus, the following categories of emergency department presentations were considered: (a) self-poisoning, (b) suicidal ideation or self-harm (without poisoning), (c) other accident and injury (excluding accidental poisoning), (d) drug and alcohol (excluding self-poisoning), (e) mental health, (f) physical illness and (g) other.

## Results

Of the 7081 individuals in the cohort, 60 individuals (male *n* = 38, 63.3%) were deceased by the study end date (Table 1). Follow-up time was missing for 910 individuals (male *n* = 408, 44.8%), of whom eight individuals were deceased (mean age at death 25.9 years, s.d. 5.7).

**Table 1:** Characteristics of deaths relative to remaining BPRR cohort

		Deceased (*n* = 60)	BPRR cohort alive (*n* = 7021)	*t*-test
Age at first presentation, mean (s.d.) years		22.17 (5.03)	19.5 (4.07)	*P* < 0.001
				**Odds ratio CI (95%)**
Gender, *n* (%)	Male	38 (63.3)	3037 (43.3)	2.34 (1.37–3.99)
	Female	21 (35)	3926 (55.9)	
**First presentation type**				
YMH clinic visit, *n* (%)		17 (28.3)	2876 (41.0)	0.80 (0.34–1.86)
ED presentation, *n* (%)		25 (41.7)	2800 (39.9)	1.21 (0.54–2.69)
Mental health unit admission, *n* (%)		8 (12.0)	216 (3.1)	5.0 (1.86–13.50)
Intensive care unit admission, *n* (%)		2 (3.0)	46 (0.6)	5.89 (1.22–28.5)
Unknown, *n* (%)		8 (12.0)	1083 (15.4)	

Gender does not include young people identifying as transgender; this is for privacy reasons owing to the small number (*n* = 59). First presentation type refers to the first visit date recorded on the linked BPRR-M&M (mortality and morbidity) database. YMH (youth mental health) clinic visit refers to clinic visits at the two BPRR clinic sites. Other primary care or mental health visits not recorded by the three linked data-sets may also have occurred. Unknown first visit type is the reference category for calculating odds ratios of first presentation types.

The median duration of follow-up from first health service visit was 7.39 years (interquartile range (IQR) 3.77 years). The total person-years of follow-up from first health service visit was 50 219. Cause of death was available for 52 individuals (*M* = 31, 59.6%). The mean age at death was 27.16 years (s.d. 6.15). The median duration of follow-up time from last health service visit and end date (either death or end of study) was 36.8 months (IQR 13.5–78.3).

### Mortality rates

Of the 60 individuals who died during the study period, 25 (42%) died owing to suicide; 19 deaths (32%) were due to accident or injury, eight (13%) deaths had a medical cause and eight (13%) deaths were of unknown cause (Table 2). The all-cause mortality rate in the BPRR cohort was 119.47 deaths per 100 000 person-years. All-cause SMR for the entire BPRR cohort was 2.0 (95% CI 1.6–2.6); the SMR for males was (5.3, 3.8–7.0), which was higher than that for females (0.91, 0.7–1.6). The highest cause-specific mortality rate was from suicide, at 49.78 per 100 000 person-years, followed by accident or injury, at 37.83 deaths per 100 000 person-years.

**Table 2:** Characteristics of deaths from suicide and accidental deaths

	Suicide (*n* = 25)	Accidental (*n* = 19)
Gender, male (%)	14 (56.0)	13 (68.4)
Age at death in years, mean (s.d.)	27.5 (6.2)	25.7 (5.8)
Time since last visit in months, mean (s.d.)	26.0 (25.7)	29.04 (21.5)
**Primary cause of death**		
Self-injury, *n* (%)	21 (84.0)	−
Hanging	15 (60.0)	−
Jumping from height	3 (12.0)	
Other	3 (12.0)	
Poisoning, *n* (%)	4 (16.0)	12 (60.0)
Transport accident, *n* (%)	−	6 (31.6)
**Secondary causes**		
Accident or injury, *n* (%)	3 (12.0)	3 (15.8)
Drug and alcohol, *n* (%)	5 (20.0)	3 (15.8)
Mental health, *n* (%)	12 (48.0)	4 (21.0)
Poisoning, *n* (%)	2 (8.0)	0
Medical, *n* (%)	0	4 (21.0)

Secondary causes are contributing causes of death other than a cause of the same type as the primary cause. Contributing causes that were related to single individuals only are not presented for privacy reasons; thus, totals may not add up. An individual could have multiple contributing causes of death.

The mean age at death was older for suicide deaths (27.5 years, s.d. 6.2), than accident or injury deaths (25.7 years, s.d. 5.8). The mean age of deaths from medical causes or where cause was under investigation was 28.4 (6.5) years. Deaths where cause was under investigation were most frequently associated with male gender (*n* = 7, 87.5%), followed by deaths from accident or injury (*n* = 13, 68.4%), suicide (*n* = 14, 56%) and medical causes (*n* = 4, 50%).

### Cause of death

The most frequent method of suicide death was hanging, accounting for 15 (60%) deaths, followed by intentional self-poisoning with four (16%) deaths (Table 2). The most common cause of accident or injury death was poisoning, which accounted for 12 (60%) deaths, followed by motor vehicle accident with six (31.6%) deaths. Medical causes included cardiac disease, neurological causes, obesity and sepsis.

Mental illness was identified as a contributing cause in 12 (48%) suicides and four (21%) accident or injury deaths. Drug and alcohol use and poisoning were secondary causes in seven (28%) suicide deaths and three (15.8%) accidental deaths. Accident or injury was a secondary cause in three (12%) suicide deaths.

### Health care use prior to all-cause mortality

Of the 60 individuals deceased from all causes during the study period, 47 (78.3%) had at least one prior presentation to the emergency department; 23 (38.3%) had a history of an emergency department presentation related to suicidal ideation, behaviour or self-harm; 24 (40%) had presented for another mental health reason; 17 (28.3%) had presented owing to drug or alcohol use; 23 (38.3%) had presented for an accident or injury; and 37 (61.7%) had presented for a medical reason (Table 3). Thirty (50%) individuals had previously had a mental health unit admission, and 12 (20%) had been admitted to a hospital ICU in the past. Ten (16.7%) had no history of either an emergency department presentation or a hospital admission.

**Table 3:** Health service use prior to mortality (all-cause)

	All-cause mortality (*n* = 60)		BPRR cohort alive (*n* = 7021)		Hazard ratio^a^ (95% CI)
	*n* (%)	No. of visits (%)	*n* (%)	No. of visits (%)	
**Emergency presentations**					
Total emergency department presentations	47 (78.3)	443 (100)	5343 (76.1)	38 031 (100)	
Suicidal ideation or behaviour	23 (38.3)	55 (12.4)	1076 (15.3)	2307 (6.1)	**2.00** (**1.02–3.91)**
Mental health	24 (40)	79 (17.8)	1763 (25.1)	5937 (15.6)	0.74 (0.37–1.46)
Accident and injury	23 (38.3)	57 (12.9)	3027 (43.1)	6957 (18.3)	**0.53** (**0.30–0.95)**
Drug and alcohol	17 (28.3)	52 (11.7)	822 (11.7)	1925 (5.1)	**1.60** (**0.80–3.21)**
Medical	37 (61.7)	178 (40.2)	4236 (60.3)	18 836 (49.5)	0.82 (0.43–1.57)
**Hospital admissions**					
Total hospital admissions		708 (100)		27 927 (100)	
Mental health unit admission	30 (50)	252 (35.6)	1474 (20.9)	8683 (31.1)	**2.79** (**1.34–5.79**)
Intensive care unit admission	12 (20)	20 (4.6)	254 (3.6)	579 (2.1)	**3.80** (**1.81–7.98)**
**No admission or presentation history**	10 (16.7)		1725 (24.6)		0.31 (0.07–1.38)

*n* refers to the number of individuals with a lifetime history of each visit or admission type. No. of visits refers to the total number of visits in the deceased or alive cohort. Mental health unit admission includes voluntary and involuntary admissions. Intensive care unit refers to admissions to the general hospital intensive care unit, not a mental health admission. Total hospital admissions includes any hospital admission type, including non-mental health admissions. Total number of mental health admissions and intensive care unit admissions are presented. Total emergency department presentations refers to the number of individuals who had at least one emergency department presentation of any type. Each emergency department presentation was only categorised as one subtype (e.g. mental health OR medical). However, an individual could have presented multiple times for a different primary presenting reason; therefore emergency department visit subtypes do not sum to 100.

Emergency department presentations and hospital admissions exclude presentations or admissions that occurred at the time of death.

a. Hazard ratios have been adjusted for gender. Transgender young people were not included in the calculations of hazard ratios owing to the small number (*n* = 59). Hazard ratios also exclude young people where follow-up time was missing (*n* = 910).

The health service visit types associated with the greatest risk of all-cause mortality were emergency department presentation for suicidal ideation, behaviour or self-harm (hazard ratio (HR) 2.00, 95% 1.02–3.91) and admission to a mental health unit (HR 2.79, 95% 1.34–5.79) or ICU (HR 3.80, 95% 1.81–7.98) (Table 3).

### Health care use prior to death due to suicide or accident

**Fig. 1:**
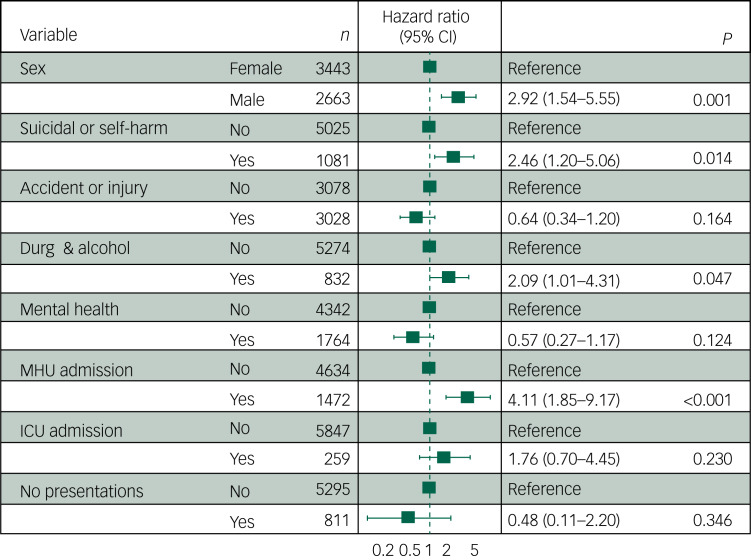
Hazard ratios for health service use and suicide- or accident-related deaths. Hazard ratio refers to the risk of suicide or accidental death relative to non-deaths. Total *N* = 6106, including 43 deaths. Excludes deaths due to medical causes (*n* = 8), young people with no final visit date recorded (*n* = 910) and people who were transgender (*n* = 59). Health visit types refer to emergency department presentations, aside from those labelled as admissions. MHU admission, mental health unit hospital admission; ICU admission, intensive care unit admission.

Table 4 presents the lifetime history of each type of health service use by cause of death. The health service visit types associated with greatest risk of death due to suicide or accident were emergency department presentation for suicidal ideation, behaviour or self-harm (HR 2.46, 95% CI 1.20–5.06), emergency department presentation related to drug or alcohol use (HR 2.09, 95% CI,1.01–4.31) and mental health unit admission (HR 4.11, 95% CI 1.85–9.17) (Fig. 1). When deaths associated with medical causes were excluded, a history of ICU admission was no longer associated with increased risk of death.

**Table 4:** Health service use prior to suicide and accidental death

	Suicide and accident mortality (*n* = 51)			BPRR cohort alive, *n* = 7021 (%) individuals
	Suicide, *n* = 25 (%) individuals	Accident and injury, *n* = 19 (%) individuals	Cause under investigation, *n* = 8 (%) individuals	
**Emergency department presentations, all**	16 (64.0)	16 (84.2)		5343 (76.1)
Suicidal ideation or behaviour	11 (44)	5 (26.3)	6 (75.0)	1076 (15.3)
Mental health	11 (44)	5 (26.3)	4 (50.0)	1763 (25.1)
Accident and injury	10 (40)	8 (42.1)	3 (37.5)	3027 (43.1)
Drug and alcohol	5 (25)	6 (24.0)	5 (62.5)	822 (11.7)
Medical	10 (40)	12 (63.2)	7 (87.5)	4236 (60.3)
**Mental health admission**	12 (48)	7 (36.8)	5 (62.5)	1474 (20.9)
**Intensive care unit admission**	2 (8)	1 (5.2)	4 (50.0)	254 (3.6)

*n* refers to the number of individuals with a lifetime history of each visit or admission type. Deaths due to medical causes (*n* = 8) are excluded. Denominators include young people of all genders and those where follow-up time was missing.

**Fig. 2:**
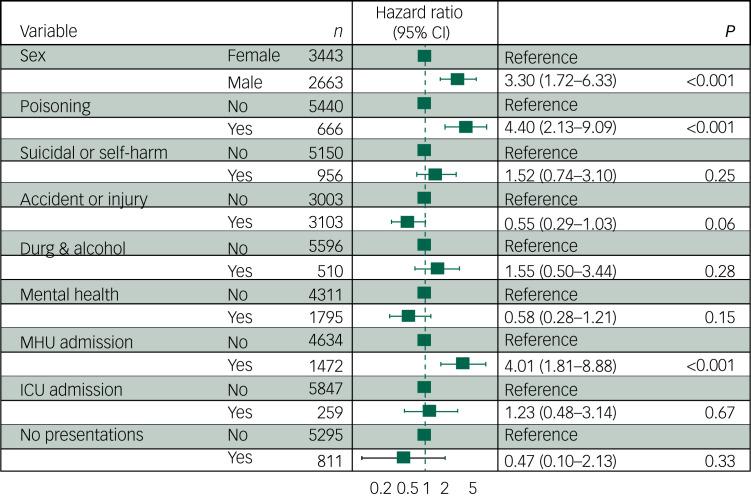
Poisoning presentations and risk of suicide- or accident-related deaths (sensitivity analysis). Results are presented of a sensitivity analysis to test the effect of categorising all self-poisoning together, regardless of whether intentional or accidental; thus, the suicidal ideation or self-harm or drug and alcohol presentations here exclude any forms of self-poisoning. Total *N* = 6106, including 43 deaths. Excludes deaths due to medical causes (*n* = 8), young people with no recorded last visit date (*n* = 910) and/or those who are transgender (*n* = 59). Health visit types refer to emergency department presentations, aside from those labelled as admissions. MHU admission, mental health unit hospital admission; ICU admission, intensive care unit admission.

The sensitivity analysis, which pooled poisonings that were intentional, accidental or where intent was unknown, found that history of presentation to the emergency department for any type of poisoning was associated with the greatest risk of death from suicide or accident (HR 4.40, 95% CI 2.13–9.09) (Fig. 2). Mental health unit admissions remained a predictor (HR 4.01, 95% CI 1.81–8.88); however, other health service use such as emergency department presentation for suicidal ideation, behaviour or self-harm, or emergency department presentation for drug and alcohol use (without poisoning), was no longer significant.

## Discussion

Premature mortality in young people accessing early intervention mental healthcare is greatly increased relative to the age-matched population. Despite the earlier stage of illness, young people in this cohort have twice the risk of death relative to the age- and sex-matched NSW population. For males in the BPRR cohort, the elevated risk of premature death increased to five times that of age-matched males in the population. This increased mortality ratio was only slightly lower than that of adults diagnosed with psychiatric disorders in in-patient or out-patient settings in a Danish register-based study of over 7 million adults.^3^ Plana-Ripoli et al (2019) reported that the mortality rate ratio (MRR) was highest among young adults and peaked at around 33 years of age (MRR 7.25, 95% CI 7.06–7.44), with the gender differential (higher in males) greatest in this period also.

Suicide was the leading cause of death in this cohort of young people accessing youth mental health services, followed closely by deaths due to accident or injury. Overall rates of suicide deaths in this cohort remain much lower than in other high-risk clinical populations, such as those discharged from psychiatric hospitals, for which a meta-analysis by Chung et al (2017) estimated a rate of 484 suicides per 100 000 person-years (95% CI 422–555).^20^ Although rates of suicide for males were double that of females in the cohort, this represented less of a gender difference than that seen in suicide rates at a population level, where rates are 3–4 times higher in males aged 15–35 years than females in the same age group.^21,22^ This is consistent with previous findings that suicide rates among psychiatric cohorts are associated with less of a gender differential than population studies.^20^ In the current study, greater all-cause mortality risk among males was driven by a higher frequency of accidental deaths and deaths where cause was under investigation. The distribution of gender in the BPRR cohort also indicates that previous findings suggesting that men are less likely to access mental healthcare^15^ are likely to apply in primary youth mental health settings.

In young people accessing early intervention youth mental healthcare, poisoning, whether intentional (suicidal) or accidental, was the single greatest primary cause of death (26.7%). When contributing causes were included, self-poisoning or substance use were primary or contributing causes in 43.3% (26/60) of all deaths. Although previous studies have identified an increased risk of death from any type of poisoning in cohorts accessing mental healthcare in adult psychiatric services or emergency departments,^2,3^ our findings confirm that such associations are present in young people accessing early intervention youth mental healthcare at earlier stages of illness. The proportion of deaths where poisoning was the primary cause also appeared to be greater in the BPRR cohort relative to other cohort studies, which report poisoning to be a primary cause in approximately 15% of deaths.^17^

Means restriction is one evidence-based suicide prevention intervention with particular relevance to poisoning deaths.^23^ Previous reviews of means restrictions targeting pharmaceuticals, including limiting pack size and restrictions on the sale of medications, have shown efficacy in reducing risk of suicide death from poisoning, as well as reduced risk of suicide death from other causes.^24^ The effects of means restriction interventions are strongest where the target is associated with the greatest lethality. Much of the evidence on means restriction of pharmaceutical drugs relates to drugs where limitations have already been introduced, for example, barbiturates, opioids or benzodiazepines, where strict limitations on prescribing are in place. Thus, it is not clear what impact further means restrictions would have in a youth cohort. The implications of restricting access to pharmaceuticals must be carefully considered in populations accessing mental healthcare, as there are already barriers to accessing adequate treatment, of which pharmacological therapy is often a key part. It is also possible that there are other indirect relationships between access to pharmaceuticals and poisoning that need to be considered in youth mental health cohorts. For example, Chitty et al (2018) previously reported that prescription of psychotropic medication was associated with reduced risk of co-ingestion of alcohol in intentional self-poisoning and thus a reduced risk of lethality.^25^

### Prior health service use and opportunities for prevention

This study confirms that knowledge of previous health service use remains a useful predictor of all-cause mortality and deaths due to suicide and accident in young people accessing early intervention services. A meta-analysis by Franklin et al (2017) reported that prior psychiatric hospital admission was associated with an odds ratio of 3.57 (95% CI 2.81–4.53) for suicide death, relative to no history of psychiatric hospital admission.^8^ A previous Danish register-based study by Hjorthøj et al (2012) also reported an increasing risk of suicide by level of psychiatric care accessed in the previous 12 months.^26^ Increasing relative risk (RR) was associated with accessing medication only (RR 5.8, 95% CI 5.2–6.6), out-patient treatment only (RR 8.2, 95% CI 6.1–11.0), emergency room treatment (RR 27.9, 95% CI 19.5–40.0) and, finally, psychiatric hospital admission (RR 44.3, 95% CI 36.1–54.4), relative to those with no psychiatric contact in the prior 12 months.

The sensitivity analysis, which combined all emergency department presentations for poisoning, whether intentional or accidental, found that previous emergency department presentation for poisoning was associated with the greatest risk of death from suicide, accident or injury among all health service visit types, including admission to a mental health unit. Previous emergency department presentations for suicidal ideation, behaviour or self-harm, where self-poisoning was not the method used, were not significant as predictors of future deaths. These findings differ from those of previous UK cohort studies, which have found emergency department presentations for self-poisoning to be associated with reduced future mortality risk relative to emergency department presentations for self-injury.^17^ On the other hand, a cohort study by Carter et al (2005) based in Australian emergency departments found a similarly elevated risk of future mortality following emergency department presentations for poisoning.^17,27^

Our results suggest that there may be further utility in providing targeted follow-up care after emergency department presentation for any type of poisoning, regardless of whether prior presentations were associated with suicidal intent. Assertive follow-up care following emergency department presentations for suicidal ideation or behaviour is an evidence-based suicide prevention intervention and is recommended practice in health systems across the world.^28^ However, these interventions do not usually extend to poisonings without suicidal intent. Accidental poisonings are strongly associated with substance use.^29^ Common treatment paradigms for drug and alcohol misuse are often based on an individual being proactive in seeking treatment.^30^ However, suicide prevention research has shown that assertive follow-up by services, even with brief contact interventions, can have an effect on reducing future suicidal behaviour.^31^ Given that substance use and alcohol dependence are strongly associated with mortality from suicide and accident across the lifespan, designing and testing interventions that integrate these approaches is likely to be worthwhile. For example, assertive follow-up and brief contact interventions that have a holistic, person-centred approach may be suitable for individuals at earlier stages of change, including those who are pre-contemplative about reducing drug or alcohol use. Ensuring that assessment and treatment of drug and alcohol use are wholly integrated into early intervention mental health services may increase the engagement of young people with this aspect of care. Such an approach would be supported by the development of clinical pathways and up-skilling of front-line workers in assessment and treatment of high-risk drug and alcohol use behaviours.

Clinical approaches to reducing premature mortality in this cohort should be combined with population-level approaches, including the development of public policy and infrastructure. For example, in Australia, increasing rates of paracetamol overdoses among young people have led the Therapeutic Goods Administration to consider increasing restrictions on the sale of paracetamol.^32,33^ The development of public digital infrastructure at a health system level is likely to be another opportunity to increase the quality and effectiveness of care and reduce adverse outcomes such as premature mortality. For example, such infrastructure could facilitate the identification of high-risk emergency department presentations and the feasibility of delivering brief contact interventions, while preserving the clinical workforce for more intensive or in-person interventions.^23^ Expanding access to other effective interventions in this clinical cohort, such as increased access to specialist mental healthcare, psychological interventions and care coordination, will also require high-quality digital infrastructure.^34,35^

### Limitations

This study had several limitations. Understanding of health service use was limited by the inclusion of only emergency department presentations and hospital admissions. Use of other health services, such as general practitioner visits or engagement with community-based mental health clinicians including psychologists or other therapists, was not measured. Greater analysis of other types of health service use, such as primary care, may have allowed for a greater understanding of engagement with services. It is important to note that the study was observational and thus not designed to test the efficacy or effectiveness of mental health services in reducing mortality outcomes. The cohort was sampled from two urban early intervention youth mental health clinics, which means young people living in rural and remote areas were not adequately represented. The study used a clinical approach to categorising emergency department visits, which may limit the external validity of our findings; however, our results were consistent with those of other research in the field.^24^

### Implications

Premature mortality in young people accessing early intervention youth mental healthcare is greatly increased relative to the age-matched population despite the early stage of illness. The elevated risk of premature mortality is especially marked for young males. Suicide was the leading cause of death in this cohort of young people accessing early intervention youth mental healthcare services, followed closely by deaths from accident or injury. Self-poisoning or substance use was a primary or contributing cause of death in almost half of deaths in this youth cohort. Previous emergency department presentation for self-poisoning, regardless of whether it was associated with intent to self-harm, was the strongest predictor of future mortality. These findings indicate that the delivery of assertive aftercare following emergency department presentations should not be limited only to presentations identified as suicidal behaviour or self-harm. More assertive patient-centred follow-up and well-coordinated specialist care are indicated for all types of self-poisoning and drug and alcohol use presentations, given the association with increased mortality.

## Data Availability

The data that support the findings of this study are available on request from the corresponding author, C.M.M., within the restrictions of the ethics approval and governance.

## References

[1] Suokas J, Suominen K, Isometsä E, Ostamo A, Lönnqvist J. Long-Term Risk Factors for Suicide Mortality After Attempted Suicide-Findings of a 14-Year Follow-Up Study. Munksgaard International Publishers, 2001. 10.1034/j.1600-0447.2001.00243.x 11473505

[2] Bergen H, Hawton K, Waters K, Ness J, Cooper J, Steeg S, Premature death after self-harm: a multicentre cohort study. Lancet 2012; 380(9853): 1568–74. 22995670 10.1016/S0140-6736(12)61141-6

[3] Plana-Ripoll O, Pedersen CB, Agerbo E, Holtz Y, Erlangsen A, Canudas-Romo V, A comprehensive analysis of mortality-related health metrics associated with mental disorders: a nationwide, register-based cohort study. Lancet 2019; 394(10211): 1827–35. 31668728 10.1016/S0140-6736(19)32316-5

[4] Australian Institute of Health and Welfare. *Deaths in Australia*. AIHW, 2020 (https://www.aihw.gov.au/reports/life-expectancy-death/deaths/data [cited 5 Dec 2020]).

[5] Kalb LG, Stapp EK, Ballard ED, Holingue C, Keefer A, Riley A. Trends in psychiatric emergency department visits among youth and young adults in the US. Pediatrics 2019; 143(4): e20182192. 10.1542/peds.2018-2192 PMC6564072 30886112

[6] Mercado MC, Holland K, Leemis RW, Stone DM, Wang J. Trends in emergency department visits for nonfatal self-inflicted injuries among youth aged 10 to 24 years in the United States, 2001–2015. JAMA 2017; 318(19): 1931–3. 29164246 10.1001/jama.2017.13317 PMC5753998

[7] Perera J, Wand T, Bein KJ, Chalkley D, Ivers R, Steinbeck KS, Presentations to NSW emergency departments with self-harm, suicidal ideation, or intentional poisoning, 2010–2014. Med J Aust 2018; 208(8): 348–53. 29669496 10.5694/mja17.00589

[8] Franklin JC, Ribeiro JD, Fox KR, Bentley KH, Kleiman EM, Huang X, Risk factors for suicidal thoughts and behaviors: a meta-analysis of 50 years of research. Psychol Bull 2017; 143(2): 187. 27841450 10.1037/bul0000084

[9] Large M, Corderoy A, McHugh C. Is suicidal behaviour a stronger predictor of later suicide than suicidal ideation? A systematic review and meta-analysis. Aust N Z J Psychiatry 2021; 55(3): 254–67. 32579030 10.1177/0004867420931161

[10] Hawton K, Bale L, Brand F, Townsend E, Ness J, Waters K, Mortality in children and adolescents following presentation to hospital after non-fatal self-harm in the multicentre study of self-harm: a prospective observational cohort study. Lancet Child Adolesc Health 2020; 4(2): 111–20. 31926769 10.1016/S2352-4642(19)30373-6

[11] Walter F, Carr MJ, Mok PL, Astrup A, Antonsen S, Pedersen CB, Premature mortality among patients recently discharged from their first inpatient psychiatric treatment. JAMA Psychiatry 2017; 74(5): 485–92. 28296989 10.1001/jamapsychiatry.2017.0071 PMC5417353

[12] Chan MK, Bhatti H, Meader N, Stockton S, Evans J, O'Connor RC, Predicting suicide following self-harm: systematic review of risk factors and risk scales. Br J Psychiatry 2016; 209(4): 277–83. 27340111 10.1192/bjp.bp.115.170050

[13] Carpenter JS, Iorfino F, Cross S, Nichles A, Zmicerevska N, Crouse JJ, Cohort profile: the Brain and Mind Centre *Optymise* cohort: tracking multidimensional outcomes in young people presenting for mental healthcare. BMJ Open 2020; 10(3): e030985. 10.1136/bmjopen-2019-030985 PMC7170572 32229519

[14] McHugh C, Song YJC, Zmicerevska N, Crouse J, Nichles A, Wilson C, Premature mortality in early-intervention mental health services: a data linkage study protocol to examine mortality and morbidity outcomes in a cohort of help-seeking young people. BMJ Open 2022; 12(2): e054264. 10.1136/bmjopen-2021-054264 PMC8860051 35190432

[15] Centre for Health Record Linkage. *How Record Linkage Works*. CHeReL, 2020 (https://www.cherel.org.au/how-record-linkage-works).

[16] Ministry of Health, NSW. *Emergency Department Data Dictionary*. Ministry of Health, NSW 2020 (https://www.cherel.org.au/data-dictionaries#section5).

[17] Bergen H, Hawton K, Kapur N, Cooper J, Steeg S, Ness J, Shared characteristics of suicides and other unnatural deaths following non-fatal self-harm? A multicentre study of risk factors. Psychol Med 2012; 42(4): 727–41. 21910932 10.1017/S0033291711001747

[18] Centre for Health Record Linkage. *NSW Mortality Data*. CHeReL, 2020 (https://www.cherel.org.au/data-dictionaries#section7).

[19] Australian Bureau of Statistics. *Regional Population by Age and Sex*. ABS, 2020 (www.abs.gov.au/statistics/people/population [cited 5 Dec 2020]).

[20] Chung DT, Ryan CJ, Hadzi-Pavlovic D, Singh SP, Stanton C, Large MM. Suicide rates after discharge from psychiatric facilities: a systematic review and meta-analysis. JAMA Psychiatry 2017; 74(7): 694–702. 28564699 10.1001/jamapsychiatry.2017.1044 PMC5710249

[21] Hill NT, Witt K, Rajaram G, McGorry PD, Robinson J. Suicide by young Australians, 2006–2015: a cross-sectional analysis of national coronial data. Med J Aust 2021; 214(3): 133–9. 33236400 10.5694/mja2.50876

[22] Australian Institute of Health and Welfare. *The Health Impact of Suicide and Self-Inflicted Injuries in Australia*. AIHW, 2019 (https://www.aihw.gov.au/reports/burden-of-disease/health-impact-suicide-self-inflicted-injuries-2019/contents/how-much-burden [updated 2021]).

[23] Robinson J, Bailey E, Witt K, Stefanac N, Milner A, Currier D, What works in youth suicide prevention? A systematic review and meta-analysis. EClinicalMedicine 2018; 4: 52–91. 31193651 10.1016/j.eclinm.2018.10.004 PMC6537558

[24] Lim JS, Buckley NA, Chitty KM, Moles RJ, Cairns R. Association between means restriction of poison and method-specific suicide rates: a systematic review. JAMA Health Forum 2021; **2**(10): e213042. 10.1001/jamahealthforum.2021.3042 PMC8727039 35977165

[25] Chitty KM, Dobbins T, Dawson AH, Isbister GK, Buckley NA. Relationship between prescribed psychotropic medications and co-ingested alcohol in intentional self-poisonings. Br J Psychiatry 2017; 210(3): 203–8. 28104739 10.1192/bjp.bp.115.172213

[26] Hjorthøj CR, Madsen T, Agerbo E, Nordentoft M. Risk of suicide according to level of psychiatric treatment: a nationwide nested case–control study. Soc Psychiatry Psychiatr Epidemiol 2014; 49(9): 1357–65. 24647741 10.1007/s00127-014-0860-x

[27] Carter G, Reith DM, Whyte IM, McPherson M. Non-suicidal deaths following hospital-treated self-poisoning. Aust N Z J Psychiatry 2005; 39(1–2): 101–7. 15660712 10.1080/j.1440-1614.2005.01515.x

[28] Hill NT, Halliday L, Reavley NJ. Guidelines for Integrated Suicide-Related Crisis and Follow-up Care in Emergency Departments and Other Acute Settings. Black Dog Institute, 2017.

[29] Lund C, Teige B, Drottning P, Stiksrud B, Rui TO, Lyngra M, A one-year observational study of all hospitalized and fatal acute poisonings in Oslo: epidemiology, intention and follow-up. BMC Public Health 2012; 12(1): 858. 23046743 10.1186/1471-2458-12-858 PMC3542203

[30] Bernstein SL, D'Onofrio G. Screening, treatment initiation, and referral for substance use disorders. Addict Sci Clin Pract 2017; 12(1): 1–4. 28780906 10.1186/s13722-017-0083-z PMC5545867

[31] Doupnik SK, Rudd B, Schmutte T, Worsley D, Bowden CF, McCarthy E, Association of suicide prevention interventions with subsequent suicide attempts, linkage to follow-up care, and depression symptoms for acute care settings: a systematic review and meta-analysis. JAMA Psychiatry 2020; 77(10): 1021–30. 32584936 10.1001/jamapsychiatry.2020.1586 PMC7301305

[32] Therapeutic Goods Administration. *Independent Review of Paracetamol Overdose*. TGA, 2022.

[33] Buckley NA, Calear A, Christensen H. Independent Expert Report on the Risks of Intentional Self-Poisoning with Paracetamol. *Therapeutic Goods Administration*, 2022.

[34] Iorfino F, Occhipinti J-A, Skinner A, Davenport T, Rowe S, Prodan A, The impact of technology-enabled care coordination in a complex mental health system: a local system dynamics model. J Med Internet Res 2021; 23(6): e25331. 34077384 10.2196/25331 PMC8274674

[35] eHealth NSW. *eHealth Strategy for NSW Health A Digitally Enabled and Integrated Health System Delivering Patient-Centred Health Experiences and Quality Health Outcomes*. 2016.

